# Long-term impact of health policy and management training for health experts from the LMICs: 2022 Dr. LEE Jong-wook fellowship program

**DOI:** 10.1093/inthealth/ihag023

**Published:** 2026-04-20

**Authors:** Vasuki Rajaguru, Jun Su Park, Soo Hyeok Choi, Sunmin Kim, Jungeun Seo, Yumin Kim, Hyejin Jung, Tae Hyun Kim

**Affiliations:** Department of Healthcare Management, Graduate School of Public Health, Yonsei University, Seoul 03722, Republic of Korea; Department of Public Health, Graduate School, Yonsei University, Seoul 03722, Republic of Korea; Department of Public Health, Graduate School, Yonsei University, Seoul 03722, Republic of Korea; Department of Public Health, Graduate School, Yonsei University, Seoul 03722, Republic of Korea; Dr. Lee Jong-wook Fellowship Programme, Graduate School of Public Health, Yonsei University, Seoul 03722, Republic of Korea; Lee Jong-wook Academy, Korea Foundation for International Healthcare, Seoul 04933, Republic of Korea; Lee Jong-wook Academy, Korea Foundation for International Healthcare, Seoul 04933, Republic of Korea; Department of Biohealth Industry, Graduate School of Transdisciplinary Health Sciences, Yonsei University, Seoul 03722, Republic of Korea

**Keywords:** capacity building, Dr. Lee Jong-wook Fellowship, health policy, Kirkpatrick model, long-term impact, training evaluation

## Abstract

**Background:**

Strengthening health leadership and management capacity in low- and middle-income countries (LMICs) is critical for building resilient health systems. The Dr. Lee Jong-wook Fellowship Program (LJWFP), implemented by the Korea Foundation for International Healthcare, was designed to equip public health administrators with competencies in policy implementation and system strengthening. However, long-term behavioural impacts of such programs are rarely evaluated beyond satisfaction and knowledge gains. This study assessed the long-term impact of the 2022 LJWFP cohort on competency achievement, satisfaction and workplace application using the Kirkpatrick evaluation framework.

**Methods:**

This mixed methods study analysed data from 13 fellows who participated in the 2022 LJWFP. Quantitative data were collected through pre- and post-training assessments, participant surveys and supervisor and peer evaluations. Qualitative evidence was obtained through follow-up assessments and structured field visits. Descriptive statistics summarized key outcomes and Pearson’s correlation analysis examined associations among competency achievement, training satisfaction and workplace application over a 2-y follow-up period.

**Results:**

The program demonstrated high training satisfaction, with substantial gains in knowledge and competency. The correlation between competency achievement and satisfaction was moderate and statistically significant (r=0.488, p<0.05), whereas the relationship between competency and field application was moderate but not statistically significant (r=0.445, p<0.05). A strong, statistically significant correlation was observed between satisfaction and field application (r=0.776, p<0.001), suggesting that participants who were more satisfied with the training were more likely to apply their learning in practice.

**Conclusions:**

The program effectively enhanced leadership skills and supported post-training applications. Future iterations should integrate mentorship and follow-up mechanisms, expand digital learning tools and measure system-level impact longitudinally. These findings highlight the value of context-tailored fellowships in advancing public health leadership and governance in LMICs.

**Acronyms**
LJWFP: Dr. LEE Jong-wook Fellowship Program
KOFIH: Korea Foundation for International Healthcare
MOH: Ministry of Health
LMICs: Low- and Middle-Income Countries
SDGs: Sustainable Development Goals
UHC: Universal Health Coverage
M&E: Monitoring and Evaluation
TOT: Training of Trainers
IR: Implementation Research
NCD: Non-Communicable Disease
HMIS: Health Management Information System
ICT: Information and Communication Technology

## Introduction

Strengthening public health leadership and governance in low- and middle-income countries (LMICs) is essential for health equity and resilient health systems.^1^ Health systems in LMICs frequently contend with enduring problems, including a shortage of human resources, weak infrastructure, insufficient regulatory frameworks and limited ability for policy implementation.^2,3^ Furthermore, a lack of structured leadership development has been identified as a major barrier to long-term healthcare system transformation.^4^ In response, investments in capacity-building programs aimed at improving policy and systems-level leadership and upskilling public health professionals have been given top priority by international health organizations, bilateral funders and technical agencies.^5,6^

The COVID-19 pandemic exposed serious gaps in emergency response, coordination and leadership across many LMICs, underscoring the urgent need for public health leaders who are strategically grounded, technically competent and actively engaged in decision-making and implementation.^7–9^ Achieving universal health coverage (UHC), strengthening emergency readiness and progressing toward the Sustainable Development Goals (SDGs) all rely on training programs that build these leadership capacities, particularly those designed and delivered within LMIC contexts.^10,11^ Capacity-building initiatives suited to the reality of low- and middle-income countries encourage local ownership, empower evidence-based policy decisions and support more accountable and inclusive health governance systems.^12–14^

Empirical research increasingly validates the effectiveness of competency-based, experiential and peer-learning models in cultivating influential leadership.^15,16^ Effective programs combine classroom learning with visits to institutions, mentoring and collaboration between countries in southern China. This helps participants learn and use what they learn in difficult situations with few resources.^17,18^ The Dr. LEE Jong-wook Fellowship Program (LJWFP), implemented by the Korea Foundation for International Healthcare (KOFIH), represents a successful model of global health capacity strengthening. Since its inception in 2007, the program has emphasized practical health policy application, health systems management, advanced technology–supported service delivery and cross-country knowledge exchange for mid- and senior-level health professionals from LMICs.^19^

Evaluation plays a central role in determining the effectiveness and long-term impact of the LJWFP, which is designed to strengthen health policy, management and leadership capacity among professionals from LMICs.^20^ Guided by the Kirkpatrick model, one of the most widely applied frameworks in global training evaluation, the assessment examines outcomes across four levels: reaction, learning, behaviour and results.^15,20,21^ Building on these immediate outcomes, the study extended the evaluation to level 3 (behaviour) by examining how fellows applied acquired knowledge and skills in their professional roles. Fellows reported implementing training concepts to improve program design, strengthen national information systems advance non-communicable disease (NCD) initiatives and enhance monitoring processes.

This study aimed to evaluate the long-term impact of the 2022 LJWFP cohort, as the 2021 cohort experienced pandemic-related disruptions that limited field-based learning. By integrating short-term learning gains with longer-term behavioural outcomes, the study sought to address a recognized gap in global health training research, where evaluations often focus on satisfaction and knowledge acquisition while overlooking post-training performance and real-world application. Using a multidimensional evaluation approach aligned with the Kirkpatrick framework, the study examined how international training programs can foster sustained leadership development and policy implementation in LMIC contexts.

## Methods

### Program overview

The KOFIH has provided a 12-week intensive health policy and management training program under the LJWFP with host institutions since 2021, targeting mid- and senior-level public health experts from LMICs (Supplementary file 1). Strengthening leadership and governance capacity is widely recognized as essential for improving health system performance and advancing UHC, particularly in resource-constrained settings.^6,10,12^ The LJWFP curriculum emphasizes leadership in health policy, governance and multisectoral coordination, which is delivered through expert-led lectures, scenario-based problem-solving, peer-to-peer learning and structured field exposure to Korean health institutions. Guided by adult learning theory, the program is designed not only to increase immediate competency gains but also to promote sustained translation of knowledge and skills into practice within fellows’ home health systems.^22–24^ To rigorously assess program effectiveness, a structured evaluation framework was applied to examine participant satisfaction, competency acquisition and post-training workplace application.^14,18,25–27^ Data were collected through pre- and post-training assessments, supervisor evaluations and follow-up surveys, contributing empirical insights into the role of structured capacity-building initiatives in strengthening public health leadership in LMICs (Supplementary Figure 1).

### Study design and participants

This study employed a mixed methods research design. The participants were chosen based on nominations from the Ministry of Health or regional health organizations to ensure representation from Asia and sub-Saharan Africa’s public-sector agencies. There were 13 fellows who participated in 2022 LJWFP and all of them were included in this study. Eligibility criteria included national or regional experience in health policy formulation, strategic planning or implementation. The program and evaluation design is presented in Supplementary Figure 2.

Ethical approval was not required, as this study formed part of an ongoing training program affiliated with the KOFIH and Yonsei University. The evaluation survey was conducted as routine program monitoring under institutional oversight and followed established ethical guidance. All procedures adhered to the principles of the Declaration of Helsinki. Written informed consent was obtained from participants prior to survey completion. The long-term impact survey was conducted during the third–fourth week of December 2024 as part of routine program evaluation activities.

### Evaluation tools and validation

Short-term effects were evaluated via a self-administered questionnaire, validated through internal consistency checks in prior studies.^28^ Reliability analysis revealed high Cronbach’s α values across key outcome domains, as presented in Supplementary Table 1. To assess sustained competency, field application and institutional behaviour changes, a structured long-term impact assessment tool was developed by one of the authors. This approach followed a three-phase methodology: literature review and qualitative interviews to identify relevant constructs; expert validation via Delphi rounds involving specialists in global health, education and public policy; and field testing and refinement with past fellows (Supplementary Table 2). The resulting tool aligned with best practices in educational measurement and health system evaluation.^29,30^

### Data collection

Data were collected via standardized, pretested tools that were given electronically at four points in time: an online survey before arriving for Korea, a supervisor/institutional needs survey before training, an early stage needs analysis (in-depth interviews upon arrival in Korea) and a post-training field visit, satisfaction and follow-up survey at the end of the program. E-mail-based structured surveys were used for long-term follow-up at 3 months and 2 years of post training (Supplementary Table 3). Focus group interviews (FGIs) and open-ended survey responses were conducted via Zoom online interim meetings and alumni exchange meetings for 2 h per country’s local time. All the data were examined through thematic content analysis to discern recurrent themes and patterns influencing the efficacy of the training. Qualitative analysis was conducted by a systematic methodology to ensure comprehensive data interpretation.

A post-training field visit evaluation was conducted in Cambodia and Laos to assess the real-world application of competencies acquired through the LJWFP 2022 Health Policy and Management course in the third week of February 2023. The evaluation team, consisting of the program director, faculty and coordinators, implemented four standardized components: a local seminar to examine action plan progress, individual interviews with fellows to review implementation status and challenges, supervisory and colleague interviews to validate workplace performance and institutional visits to observe operational contexts. In Cambodia, activities included a seminar, two fellow interviews and supervisory consultations at Phnom Penh Municipal Referral Hospital. In Laos, the team conducted a seminar, four fellow interviews including three former fellows and institutional assessment at the Maternal and Newborn Hospital in Vientiane. All observations, interview notes and institutional findings were systematically documented to generate qualitative evidence of implementation and behavioural outcomes.

### Data analysis

The quantitative data were analysed using SPSS Statistics for Windows version 24.0 (IBM, Armonk, NY, USA). Descriptive statistics (mean, standard deviation [SD], range) were computed for continuous variables such as pre- and post-training competency scores, satisfaction and field application metrics. Categorical variables (e.g. sex, country, department) were summarized using frequencies and percentages. Paired sample t-tests were conducted to assess changes between pre- and post-training scores across subject modules and correlation analysis (Pearson’s r) was performed to examine relationships among competency, satisfaction and workplace behaviour indicators. Subgroup comparisons by sex, region and department were also explored using appropriate parametric tests. All the open-ended responses were systematically reviewed and coded by inductive thematic analysis. The analysis process involved multiple stages: initial familiarization with the data, generation of initial codes, identification of themes, review and refinement of themes and core learning and subthemes related to training effectiveness and long-term impact. Coding and thematic organization were conducted using NVivo version 2.0 (Lumivero, Denver, CO, USA). A p-value <0.05 was considered statistically significant.

## Results

### General characteristics of the participants

Table 1 presents the 2022 cohort of LJW Fellows (N=13) representing a diverse group of mid-career health professionals from LMICs, with an average age of 38.6±7.4 y. A majority were male (69.2%) and the regional split showed 61.5% were from Africa and 38.5% from Asia. Most of the participants were affiliated with a ministry of health (69.2%) and other government health agencies such as public or regional hospitals or research institutions. The areas of specialization were human resource (HR) management and training (30.8%) and chronic disease management (23.1%). In terms of job roles, 30.8% were general administrators, 23.1% were senior executives and 23.1% were medical staff. The average professional experience was 10.4±5.9 y, with nearly half (46.2%) having >10 y of service. The survey response rate was exceptionally high, averaging 99.3%, indicating strong participant engagement.

**Table 1.** General characteristics of the participants (N=13).

Characteristics	Values
Age (years), mean±SD	38.6±7.4
Age group (years), n (%)	
<35	4 (30.8)
35–39	5 (38.5)
≥40	4 (30.8)
Gender, n (%)	
Male	9 (69.2)
Female	4 (30.8)
Continent, n (%)	
Asia	5 (38.5)
Africa	8 (61.5)
Country, n (%)	
Ghana	2 (15.4)
Laos	1 (7.7)
Other Southeast Asia (Mongolia, Vietnam, Indonesia, Cambodia)	3 (23.1)
Ethiopia	2 (15.4)
Uganda	2 (15.4)
Uzbekistan	1 (7.7)
Tanzania	2 (15.4)
Affiliated organization, n (%)	
Government health agencies	9 (69.2)
Public hospitals/research institutes	4 (30.8)
Department/main field, n (%)	
Maternal and child health	2 (15.4)
Chronic disease management	3 (23.1)
Health information systems	2 (15.4)
Health education	1 (7.7)
Disease prevention and health promotion	1 (7.7)
Health administration	0 (0.0)
HR management and training	4 (30.8)
Job position, n (%)	
Senior executive	3 (23.1)
Middle manager	2 (15.4)
General administrator	4 (30.8)
Specialist/professional	0 (0.0)
Medical staff	3 (23.1)
Technical staff	1 (7.7)
Years of experience, mean±SD	10.4±5.9
Years of experience (years), n (%)	
<7	4 (30.8)
7–9	3 (23.1)
10–14	3 (23.1)
≥15	3 (23.1)
Survey response rate (%), mean±SD	99.3±2.6

### Program evaluation of the fellowship program

Table 2 illustrates strong performance across all evaluated domains of the training program. The average HR input score was rated high (95.4±12) and pre-training preparation processes, including application support, immigration assistance, orientation and briefing sessions, also received strong evaluations. Knowledge and competency showed a marked improvement, increasing from pre-training (2.8±0.9) to post-training (4.6±0.3). Training satisfaction was exceptionally high, with perfect scores for clarity of objectives, safety support, logistics, willingness to participate again and cultural awareness, alongside strong ratings for content quality (94.5±4.7) and training environment (96.1±5.4). Job continuity reached 100% and workplace application of action plans received positive supervisor (73.3±2.8) and peer (71.9±5.6) assessments. Long-term engagement remained strong, with high levels of continued exchange (90.8±11.9) and follow-up participation (94.8±10.1), demonstrating the program’s sustained impact.

**Table 2.** Program evaluation of the domains by Kirkpatrick’s four-level evaluation.

Evaluation model	Domain	Variable	Mean±SD
Reaction	Human resource	Human resource inputs	95.4 ± 12
	Pre-training preparation process	Application and selection support	94.6 ± 9.7
		Arrival and immigration support	97.1 ± 4.8
		KOFIH orientation session	91.2 ± 12.5
		Training institute briefing	98.7 ± 3.2
Learning	Knowledge and competency scores	Before training	2.8 ± 0.9
		After training	4.6 ± 0.3
	Training satisfaction	Clarity of training objectives	100 ± 0
		Training content quality	94.5 ± 4.7
		Training environment	96.1 ± 5.4
		Safety support by training institutions	100 ± 0
		Support from arrival/departure agencies	100 ± 0
		Willingness to participate again or recommend to colleagues	100 ± 0
		Awareness of Korea	100 ± 0
Behaviour	Job continuity	Job continuity	100 ± 0
	Action plan workplace application	Supervisor evaluation	73.3 ± 2.8
		Peer evaluation	71.9 ± 5.6
	Continuity of exchange	Fellows’ ongoing exchange	90.8 ± 11.9
		Post-program follow-up exchange	94.8 ± 10.1

### Pre- and post-training evaluation of curriculum

Figure 1 illustrates substantial improvements in participants’ self-assessed competencies across 20 subject areas following the training program. Pre-training mean scores, which ranged from 2.0 to 3.4, indicate relatively low baseline knowledge, especially in Korean language education (2.0), research methodology (2.1) and epidemiology (2.5). After training, scores improved dramatically, with post-training means ranging from 3.8 to 5.0, showing gains of >2 points in many areas. The highest post-training score was observed in developing an action plan (5.0), followed by healthcare project management (4.9) and health planning and programs (4.9).

**Figure 1.**
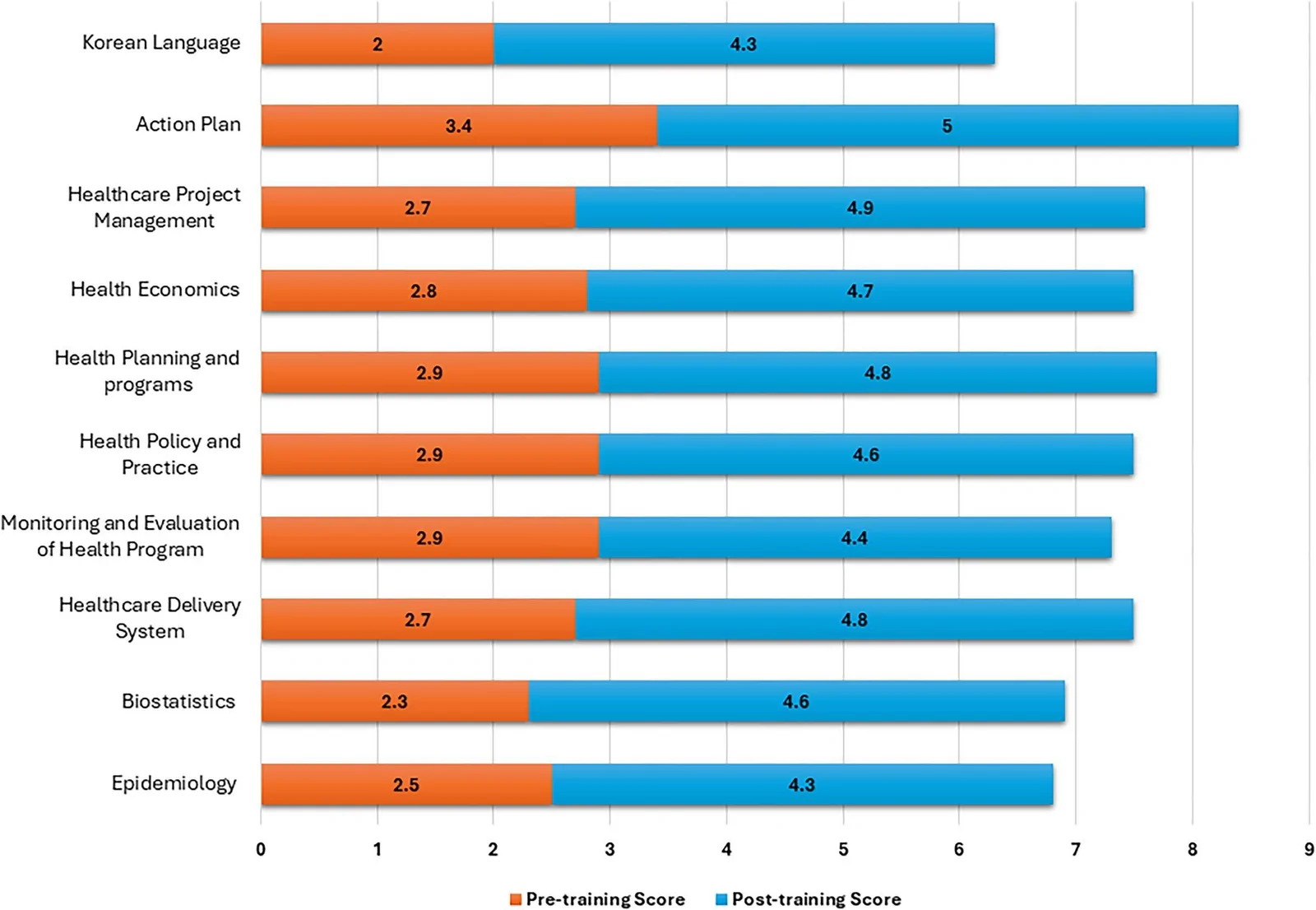
Mean differences in pre- and post-training evaluation of curriculum. Scores are measured on a 5-point Likert scale (1=very low, 5=very high). Pre-training scores reflect fellows’ self-assessed baseline competency at program entry and post-training scores represent self-evaluated competency immediately upon course completion. Higher post-training scores across all modules indicate substantial improvement in knowledge and skills gained during the 2022 LJWFP.

### Short-term impact of the fellowship program

Table 3 highlights the program’s strong performance across evaluation phases. Pre-training preparation was highly rated (94.7%) and knowledge improvement during training exceeded expectations. Post-training, satisfaction reached 96.4, competency improvement 93.8 and resource adequacy 96.5. At the 3-month follow-up, job continuity achieved a perfect score (100%) and ongoing exchange activities were maintained at 92.8%.

**Table 3.** Performance indicator measurement of mid-term and short-term impact of fellowship.

Evaluation model	Evaluation period	Performance indicator	Target score^a^	Actual score
Results	Upon completion of training (0–1 month after)	Training satisfaction	90	96.4
		Competency achievement and improvement	90	93.8
		Adequacy of physical resource inputs	80	96.5
		Adequacy of human resource inputs	80	95.4
	Post-training impact (3 months after)	Professional outcomes		
		Job continuity	80	100
		Continuity of exchange program and network building and KGA activities	90	92.8

KGA: KOFIH global alumni.

a 100-point scale.

### Long-term impact of the fellowship program

Table 4 presents the correlation analysis results controlling for age, sex, affiliation and department, revealing that competency achievement is moderately and positively associated with satisfaction (r= .488, p<0.05) and field application (r=0.445, p=0.115). In contrast, the relationship between satisfaction and field application was strong and statistically significant (r=0.776, p<0.05) (Supplementary Figure 3).

**Table 4.** Correlation of competency achievement, satisfaction and field application by adjusting covariates.

Evaluation model	Key variables	Competency achievement	Satisfaction	Field application
Results (long-term impact, 2 y after)	Competency achievement	1.000	0.488*	0.445
	Satisfaction		1.000	0.776**
	Field application			1.000

*p<0.05, **p<0.001.

Figure 2 shows significant relationships between competency, satisfaction and behavioural outcomes. Supervisor- and co-worker-rated work improvement were strongly correlated (r=0.57, p=0.02 and r=0.80, p=0.04, respectively). Self-perceived competency was positively associated with satisfaction (r=0.64). Competency improvement was negatively correlated with self-perceived competency (r=−0.63, p=0.01) and self-rated work improvement (r=−0.56, p=0.02), suggesting increased awareness of growth needs. Field application demonstrated moderate positive associations with satisfaction (r=0.38, p=0.05) and competency achievement (r= 0.42, p=0.001).

**Figure 2.**
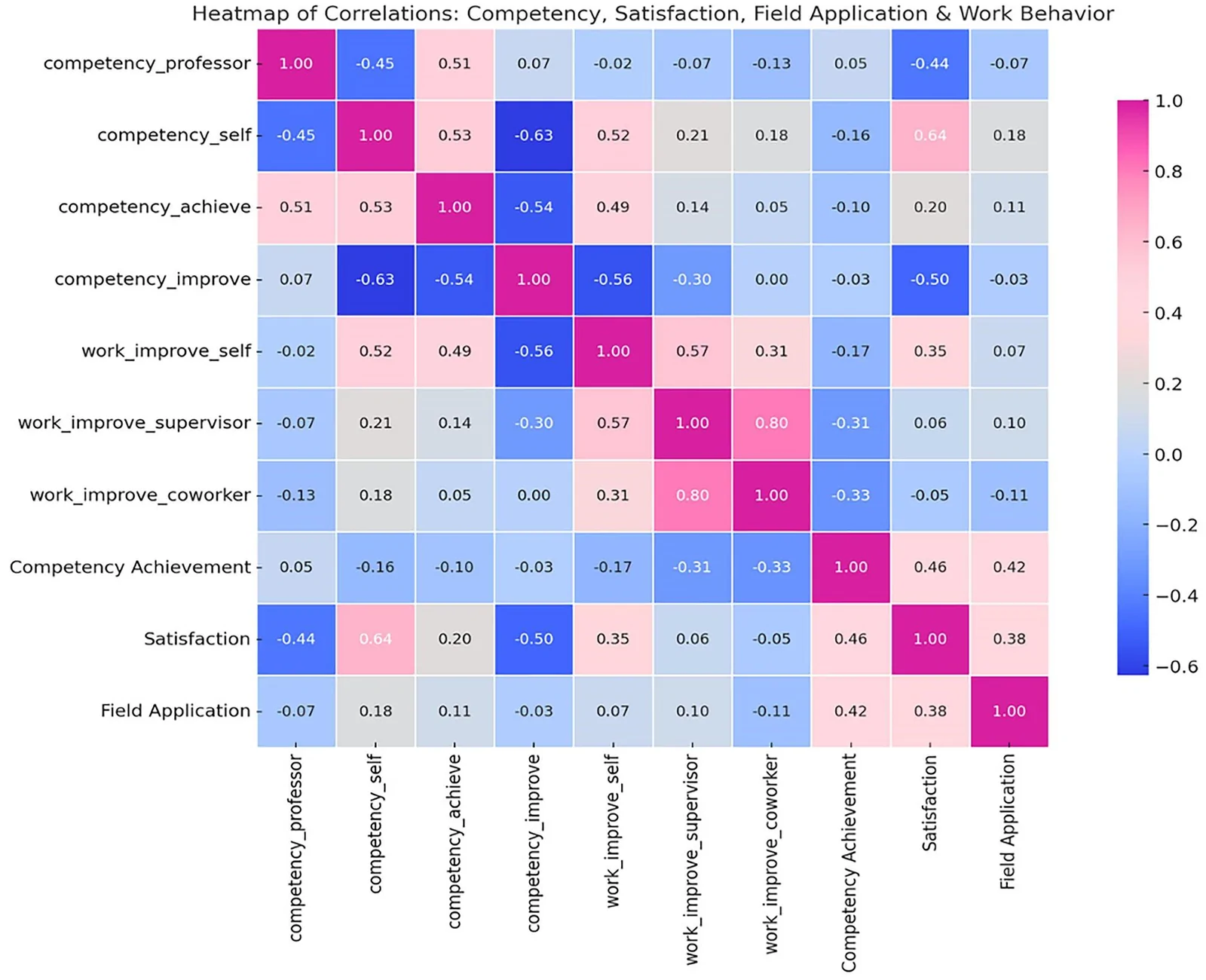
Heatmap of correlation: competency achievement, satisfaction, field application and workplace engagement. Warmer colours indicate positive associations and cooler colours indicate negative associations. Stronger correlations were observed between workplace improvement ratings (self, supervisor, coworker) and between satisfaction, self-rated competency and field application.

### Action plan development and implementation progress of the fellows

Supplementary Table 4 provides a clear snapshot of how fellows from each country developed and carried out their action plans after the fellowship. It summarizes the health challenges they prioritized, the goals they set and the practical steps they took, from conducting needs assessments and creating training materials to introducing digital tools, strengthening services and engaging communities. The results also note the progress made in each setting and show how these efforts align with national priorities and relevant SDGs. Together, it illustrates how fellows translated their learning into real-world improvements and offers early signs of meaningful change within their local health systems.

### Field visits outcomes

Findings from the field visits demonstrated active implementation of action plans and meaningful workplace application of fellowship-acquired skills. Cambodian fellows (n=2) reported progress in NCD service improvements, digital health usage and patient education initiatives, with supervisors noting enhanced leadership and program coordination. In Laos, fellows (n=4), including former 2021 fellows, demonstrated uptake of monitoring tools, strengthened referral and reporting workflows and improved alignment with national health policy directives. Seminar discussions in two countries revealed increased confidence in policy analysis (89%), strengthened team communication (92%) and the emergence of interdepartmental collaboration linked directly to fellowship training. Supervisors corroborated fellows’ self-reported outcomes, indicating clear improvements in professional behaviour, organizational performance and the practical translation of training into operational contexts (Supplementary Table 5).

### Qualitative approach for FGI results

The thematic analysis showed that the 2022 LJWFP produced broad and lasting impacts across the individual, organizational and system levels. Fellows reported strengthened leadership capacity, greater confidence in managing national programs and improved understanding of health system operations. The technical skills gained during training, such as NCD management, digital tools, laboratory systems and data monitoring, were directly applied in the workplace, demonstrating strong skill transfer. Exposure to Korea’s work culture enhanced teamwork, communication and implementation practices, while cultural immersion reinforced professionalism, respect and accountability. Many participants also adopted digital innovations, developing new electronic systems and dashboards in their home settings (Supplementary Table 6 and Supplementary Figure 4).

## Discussion

The findings of this study indicate that the LJWFP has achieved meaningful improvements across multiple evaluation domains, including knowledge acquisition, competency development, training satisfaction and field application. The knowledge and competency scores increased during training, while satisfaction levels consistently exceeded 90% across categories such as content quality, logistical support and willingness to recommend. These results align with Kirkpatrick’s four-level training evaluation model, particularly demonstrating success in the ‘learning’ and ‘behaviour’ levels.^21^ Comparable global leadership training programs such as the Emerging Voices for Global Health and the TDR Global Health Leadership Initiative also reported significant self-efficacy and behavioural change after structured competency-based training.^22,31,32^

More notably, the correlation findings revealed that participant satisfaction was strongly associated with real-world field application, reinforcing the program’s ability to translate theoretical knowledge into practical workplace impact. This echoes outcomes from similar capacity-building programs in Korea and abroad, including the Korea International Cooperation Agency training programs and Seoul National University’s Global Health Security Fellowship, which highlighted that high learner satisfaction and contextual relevance were key predictors of post-training institutional engagement.^33,34^ Similarly, the World Health Organization (WHO) Western Pacific Regional Office emphasizes such field application as an indicator of leadership training success.^35^ The high level of workplace application also validates the LJWFP’s focus on individualized action plans and their alignment with institutional needs, a feature increasingly recommended in global leadership development models.^24^

The program’s long-term impact was also reflected in the high level of continued exchange and sustained job retention among fellows. Beyond strengthening technical skills, the fellowship appeared to cultivate lasting professional relationships and a deeper sense of commitment to their institutions and national health systems. This sustained engagement aligns with Korean ODA policy priorities that emphasize post-training sustainability and the broader ripple effects of capacity building across regions.^36^ Compared with traditional short-course training programs, which often fail to maintain engagement after completion,^37^ the LJWFP’s ongoing network-building and peer engagement reflect a mature and well-integrated model. The use of peer evaluations, institutional feedback and a dedicated alumni network adds further evidence of its systemic and sustainable impact on LMIC health systems.

Our study also showed a negative correlation between perceived growth needs and self-confidence, particularly between competency improvement and self-perceived competency. This finding suggests that as fellows strengthened their knowledge and skills, they became more aware of their remaining limitations and areas requiring further development. Rather than reflecting reduced confidence, this pattern may indicate increased professional maturity and critical self-reflection an important characteristic of effective public health leadership.^26,38^ It underscores the need for ongoing mentorship, personalized feedback mechanisms, and a robust follow-up system beyond initial training. Integrating digital learning platforms, regional hubs and competency tracking systems as recommended in the WHO’s Global Competency Framework^3^ could further strengthen future iterations of the LJWFP and ensure its scalability and policy relevance.

The post-evaluation field visits provided valuable insights into the long-term behavioural and institutional impact of the LJWFP, demonstrating that competencies gained during the program translated into meaningful workplace contributions in LMIC settings. Fellows applied leadership, analytical and policy skills to advance ongoing programs, strengthen operational processes and improve coordination across teams and sectors.

The thematic findings suggest that the 2022 LJWFP had a meaningful and lasting impact on fellows’ leadership confidence, technical skills, digital innovation and professional growth. Participants described not only gaining new knowledge but also developing a stronger sense of identity and responsibility as public health leaders—an outcome consistent with findings from the global health leadership literature. The observed improvements in leadership and decision-making capacity echo broader evidence showing that well-structured training programs can enhance strategic thinking and organizational performance in LMIC settings.^4,13^ The practical transfer of technical skills into workplace improvements is consistent with studies showing that competency-focused training models, such as Korea’s Health Management Information System, NCD and digital-health approaches, enhance system efficiency and data quality when localized appropriately.^30,39^ Fellows’ adoption of digital tools and interest in health information systems also reflect global trends toward digital transformation as a foundation for UHC.^10^ The role of cultural immersion in shaping values of punctuality, respect and safety mirrors findings that experiential learning strengthens professional identity and promotes adaptive leadership.^22,37^ Finally, the emergence of sustained peer networks and post-program collaboration reinforces literature highlighting the importance of ongoing professional communities for maintaining momentum in health system reforms.^6,7^ Overall, these themes demonstrate that the fellowship contributed not only to individual skill development but also to broader system-level strengthening supporting international calls for transformative capacity-building models in global health.^12,40^

The overall strength of the program lies in its competency-based design, grounded in adult learning principles and aligned with global health system needs. The LJWFP demonstrated strong performance across preparation, delivery and post-training outcomes, offering a well-structured learning experience for fellows from diverse LMICs. Interactive modules, expert instruction and field exposure effectively strengthened public health policy and management competencies, reflected in high participant satisfaction and improved workplace performance. The continued application of action plans and active post-program engagement further underscore the training’s real-world relevance. These results parallel global evidence supporting contextualized, experiential and competency-driven leadership development as essential for strengthening health system resilience and advancing progress toward the SDGs in LMICs.

This study has several limitations. First, the sample size was relatively small, with only a limited number of fellows from the 2022 cohort participating in the evaluation, which may restrict the generalizability of the findings to all LMICs or fellowship alumni. The small sample also limited statistical power, and although exact p-values were reported, confidence intervals were not emphasized due to the exploratory nature of the analysis. Findings were therefore interpreted with greater emphasis on effect sizes and directionality rather than statistical significance alone. Second, the study primarily relied on self-reported data and post-training satisfaction surveys, which may be subject to desirability bias and recall limitations. Third, while short-term and mid-term impacts were assessed, longer-term institutional or population-level health outcomes influenced by the fellows’ actions were not measured. Finally, the absence of a control group or baseline comparison in some domains limits the ability to attribute observed changes solely to the intervention.

### Future directions

Future evaluations of the program should adopt a longitudinal mixed methods approach, incorporating qualitative interviews, workplace observations and objective indicators of policy or systems change influenced by fellows from 2023 to 2025. Expanding the cohort size and integrating data from multiple years would enhance the statistical power and offer comparative insights across contexts. Furthermore, building an alumni tracking system to monitor career progression, policy contributions and institutional change over time can strengthen the evidence base for sustained capacity building. Collaborations with national ministries and institutions could facilitate follow-up studies that link training outcomes to broader health system performance metrics.

## Conclusions

The LJWFP demonstrated strong effectiveness in enhancing competencies, training satisfaction and the real-world application of public health knowledge among policy and management professionals from LMICs. The program’s strengths lay in its tailored curriculum, immersive and practice-oriented training design and sustained post-training engagement. Beyond individual competency gains, the findings suggest meaningful contributions to strengthening LMIC healthcare delivery systems through improved leadership, program implementation and institutional coordination. By enriching the knowledge base and practical capacity of public health experts, the fellowship supported broader workforce development and reinforced national capacity-building efforts. Despite inherent limitations, the evaluation provides encouraging evidence of the program’s role in advancing health policy leadership and promoting system-level improvements. With continued investment, refinement and rigorous longitudinal follow-up, this model holds strong potential for scalability and replication across global health capacity-building initiatives.

## Supplementary Material

ihag023_Supplemental_File

## Data Availability

The data are available from the corresponding author upon reasonable request.
