# Supplementary material for: Long-term impact of health policy and management training for health experts from the LMICs: 2022 Dr. LEE Jong-wook fellowship program
Source: Int Health. 2026 Apr 20;18(6):877–86. doi: 10.1093/inthealth/ihag023 (PMC13728689; doi:10.1093/inthealth/ihag023)
Supplement: Supplementary file 1 [file ihag023_supplemental_file.docx]

**Supplementary appendices**

The selected institutions typically administer the same specialized training course for two to three consecutive cycles. This continuity allows for iterative curriculum refinement and programmatic improvement based on accumulated experience. Training institutions fulfill multiple critical functions beyond basic instruction, including candidate assessment through interviews, development of detailed field-specific curricula tailored to participant needs, delivery of comprehensive follow-up support, and collaborative evaluation of training outcomes in partnership with KOFIH.

**Program Title**

The Dr LEE Jong-wook Fellowship Program, Training Course for Health Policy Administration and Management

**Objectives**

- To train public health professionals from developing countries in the field of healthcare management and finance so that they can manage national policies and health related programs effectively as well as help set up and implement good health-related policies in their home countries
- To build partnership with developing countries for the possible expansion of Korean public health institutions into the countries
- To share Korea’s experience in overall healthcare management and policy making procedures and even disseminate Korean culture to developing countries

**Program Implementation Stages and Responsibilities**

The Dr. LEE Jong-wook Fellowship Program follows a structured, three-phase workflow involving multiple stakeholders: KOFIH (Korea Foundation for International Healthcare), designated training institutions, partner countries, and the fellows themselves. Each phase is designed to ensure clarity in responsibilities, alignment with program objectives, and sustainability in post-training impact.

1. Pre-Training Preparation: KOFIH selects host institutions and coordinates with partner countries, while institutions prepare training materials and partner ministries nominate eligible candidates.
2. Training Implementation: Host institutions deliver academic content and practical modules; fellows participate in structured activities and are supported by academic and mentoring teams. KOFIH ensures training quality and provides diplomatic and technical support.
3. Post-Training Follow-up: Institutions conduct assessments and offer continued academic support. Fellows are encouraged to apply their learning in home country settings, while KOFIH sustains alumni networks and evaluates program impact.

**Figure** S1. **Dr. Lee Jong-wook Fellowship** programme **conceptual framework. KONEPS, Korea Online E-Procurement system**; **KOFIH, Korea Foundation for International Healthcare**.

**Figure S2. Program design: Evaluation model related outcome measures and timeline**

**Table S1. Tool development and validation**

| Indicators | Tool (Likert scale) | No. Of items (subcategories) | Cronbach’s alpha | Reliability/ Appropriateness |
| --- | --- | --- | --- | --- |
| Training satisfaction | 5 points | 7 (33) | *0.771* | *Highly reliable* |
| Knowledge and competency improvement | 5 points | 8 (20) | *0.784* | *Highly reliable* |
| Competence achievement/ improvement | 10 points | 2 (3) | *0.857* | *Highly reliable* |
| Work enhancement/ job adoption of learned knowledge | 5 points | 2 (10) | *0.805* | *Highly reliable* |

**Table S2. Process of long-term impact tool development**

| Tool Development | Measurement Target | Assessment Time | Metrics |
| --- | --- | --- | --- |
| Tools developed, tested, and modified | Demographic Information | Assessed during the admission process | Country, Gender, Position |
|  | Short-term Change | Evaluated via immediate feedback | Achievement, Satisfaction, Action Plan implementation |
|  | Mid-term Achievement | Assessed within six months post-graduation | Workplace Application, Progress of action plan, Career Promotion |
| Tool tested | Long-term Impact | Analyzed through surveys conducted over two years post-graduation | Competency Achievement Training Satisfaction Field Application |

**Table S3. Evalaution process and duration**

| Assessment | Timing | Method | Purpose |
| --- | --- | --- | --- |
| Pre-departure | August 2022  (1st week) | Online Survey | Identify learning priorities and contextual needs |
| Training period  (Supervisor & host institution Feedback) | August 2022 (2nd week) | Online Survey | Ensure alignment with national and organizational objectives |
| End of the training and 1month after training Follow-up | October 2022 (1st week) | In-depth Interview  Zoom interim meeting | Compare curriculum-based knowledge and competency achievement, Satisfaction, Action Plan implementation |
| Short term impact (3months) | December 2022 (3rd week) | Online Survey | Workplace Application, Progress of action plan, Career Promotion |
| Long term impact (2 years) | December 2024  (2^nd^ week) | Online survey  Zoom -Network building exchange meeting | Competency Achievement Training Satisfaction Field Application |

**Figure S 3. Long term impact of competency, satisfaction and field application**

|  | Competency Achievement | Satisfaction | Field Application |
| --- | --- | --- | --- |
| 1 | 93.3 | 97.5 | 93.3 |
| 2 | 96.7 | 100 | 100 |
| 3 | 98.3 | 96.1 | 84.4 |
| 4 | 93.3 | 97.5 | 93.3 |
| 5 | 96.7 | 100 | 100 |
| 6 | 98.3 | 99.6 | 100 |
| 7 | 100 | 99.3 | 100 |
| 8 | 100 | 98.2 | 84.4 |
| 9 | 76.7 | 90.9 | 84.4 |
| 10 | 98.3 | 88.4 | 95.6 |
| 11 | 96.7 | 94 | 97.8 |
| 12 | 100 | 98.9 | 95.6 |

**Table S4.** Description of the Action plan development and implementation progress

| Country | Project Title | Primary Goal | Key Activities | Lead Department | SDG(s) | Progress |
| --- | --- | --- | --- | --- | --- | --- |
| Uzbekistan | Improving hemodialysis services | Improve dialysis quality via PPP | Develop PPP tender; partner with international providers; establish modern centers | MoH – PPP Dept. | 3 | Center approved; renovation completed; free services started |
| Uganda | COVID-19 vaccine communication and mobilization | Increase uptake & reduce hesitancy | Media sensitization; community outreach; influencer engagement | MoH – Health Promotion | 3, 10 | Pilot underway; pending financial clearance |
| Mongolia | Strengthening lab diagnostics workforce | Improve diagnostics capacity | Needs assessment; targeted training; skills monitoring | National Centre for Public Health | 3 | Protocol submitted |
| Uganda | Strengthening HMIS for NCDs | Improve NCD data quality | Develop NCD curriculum; national/regional training; integrate indicators | MoH – NCD Dept. | 3, 10 | Ongoing |
| Ethiopia | End-to-end visibility for pharmaceutical supply chain | Establish national PIS | TWG formation; system assessment; dashboard development | MoH Ethiopia | 3 | Benchmarking pilot ongoing |
| Ethiopia | Reducing neonatal mortality at Bonga Hospital | Reduce neonatal deaths | Procure NICU equipment; renovate wards; provider training | Bonga General Hospital | 3, 12 | Renovations completed; equipment installed |
| Ghana | Improving neonatal mortality & stillbirth data | Improve MNCH data accuracy | Staff training; data analysis; reporting accuracy checks | Ghana Health Service | 3 | Pilot completed in 2 hospitals |
| Ghana | Attracting and retaining rural health workers | Attract & retain rural clinicians | Retention needs assessment; FGDs; policy framework development | MoH Ghana | 3, 10 | In progress |
| Laos | Building capacity for international cooperation | Strengthening IC for UHC & SDGs | Consultations; training; joint M&E | MoH – Int’l Cooperation Division | 17 | In progress |
| Tanzania | Reforming CPD system | Strengthening CPD frameworks | Workshops; MoUs; M&E system development | Medical Council of Tanganyika | 3 | CPD research proposed |
| Tanzania | Ethical training for nurses & midwives (INEPT) | Improve respectful care | RCC training; supervision; exit interviews | MoH – Nursing Directorate | 3, 16 | Protocol submitted |
| Cambodia | Integrated HTN & diabetes management | Improve NCD management | Staff training; check-up packages; digital tools | Phnom Penh Referral Hospital | 3, 9 | Digital tools deployed, scaling ongoing |
| Cambodia | Digital transformation of health records | Implement EHR in 15 centers | ICT training: laptops provided; pilot & scale-up | Preah Sihanouk Provincial Health Dept. | 3, 9 | EHR piloted in 5 centers |

PPP = Public–Private Partnership; MoH = Ministry of Health; NCD = Non-Communicable Disease; HMIS = Health Management Information System; PIS = Pharmaceutical Information System; TWG = Technical Working Group; NICU = Neonatal Intensive Care Unit; MNCH = Maternal, Newborn, and Child Health; FGD = Focus Group Discussion; IC = International Cooperation; UHC = Universal Health Coverage; SDGs = Sustainable Development Goals; CPD = Continuous Professional Development; INEPT = Improving Nurses and Midwives Adherence to Ethical Principles Through Training; RCC = Respectful and Compassionate Care; HTN = Hypertension; EHR = Electronic Health Record; M&E = Monitoring and Evaluation; Dept. = Department; Int’l = International.

**Table S5.** Description of the Action plan development and implementation progress

| Component | Cambodia (2022) | Laos (2022) | Purpose |
| --- | --- | --- | --- |
| Local Seminar | Seminar with fellows, supervisors, colleagues, and KOFIH office | Seminar with MOH officials, fellows, supervisors, colleagues | Share implementation progress, identify challenges, strengthen network |
| Fellow Interviews | 2 fellows (2022 cohort) | 3 fellows (2021) + 1 fellow (2022) | Assess action plan implementation, challenges, support needs |
| Supervisor Interviews | 2 supervisors | 1 supervisor per fellow | Validate performance change and behavioral outcomes |
| Colleague Interviews | 3 colleagues | 1 colleague per fellow | Assess team-level impact and workplace changes |
| Institutional Visit | Phnom Penh Municipal Referral Hospital | Mother and Newborn Hospital, Vientiane | Assess operational context & feasibility of action plan application |
| Key Findings | Improvements in NCD management, digital health use, patient education | Strengthened monitoring, referral coordination, policy implementation practices | Evidence of behavioral change and improved workplace performance |

**Table S6. Qualitative analysis of Thematic responses by the 2022 fellows**

| Theme | Core learning | Quotes |
| --- | --- | --- |
| Strengthened health-system leadership capacity | Fellows reported increased confidence in designing and coordinating national or regional health programs. They identified improved skills in problem-solving, stakeholder engagement, performance monitoring, and evidence-informed decision-making. Many emphasize applying Korean program management models to streamline processes. | “Before the fellowship, I managed projects. Now I *lead* them. I can justify decisions with evidence and communicate clearly with my ministry leadership.” (U1) |
| Enhanced technical competencies with practical application | Training in NCD management, HMIS, digital health, laboratory systems, and supply-chain improvements translated into actual workflow enhancements. Fellows adapted Korean tools (dashboards, SOPs, quality indicators) to local systems. | “The tools we practiced in Korea became templates for my national guidelines. It wasn’t theory I applied it the moment I returned.” (M2) |
| Expanded interprofessional collaboration | Fellows described stronger teamwork, improved clarity of roles, appreciation for multidisciplinary practice, and more intentional communication between clinical, administrative, and public-health units. | “I now involve IT, clinicians, and admin teams from day one. Korea showed me that collaboration isn’t optional it’s the system.” (E1) |
| Improved program implementation and monitoring skills | Fellows applied Korean-style monitoring, supervision, and quality-improvement methods. They introduced periodic audits, simplified data tools, and clearer reporting pathways. | “Using the Korean QI model, we created a simple monitoring checklist. Our reporting rate increased instantly.” (G1) |
| Cultural influence on professional identity | Participants highlighted how cultural experiences instilled values of respect, punctuality, safety culture, and structured communication. Exposure to Korea’s organizational discipline shaped their leadership mindset. | “Korean culture taught me discipline and respect in daily work—small behaviors that create a high-quality system.” (T2) |
| Shift in perception toward digital health and innovation | Fellows gained a deeper appreciation for digital transformation after observing Korea’s integrated health information systems. This encouraged adoption of EHRs, dashboards, and electronic reporting in home settings. | “Seeing Korea’s digital health system convinced me we must digitize. Now our team is piloting an EHR in two districts.” (C3) |
| Strengthened global health networks | Fellows continued exchanging expertise through post-program communication groups, joint projects, and mentorship. Some initiated bilateral collaborations with Korean institutions. | “Our WhatsApp group still shares resources weekly we continue learning from each other long after the program ended.” (L1) |
| Professional empowerment | Many fellows reported renewed motivation, clearer career aspirations, and enhanced credibility within their ministries. The fellowship was perceived as a catalyst for accelerated professional growth. | “This program elevated my position in the ministry. My supervisors trust me with national tasks now.” (U3) |

U= Uganda; M=Mongolia; C= Cambodia; G=Ghana; T=Tanzania; E=Ethiopia; L=Laos

**Figure S4.** Thematic structure of the fellows’ experience throughout the long term follow up-
